# Estimation of Demand and Supply Balance of Livestock Feed in Haru District, Western Ethiopia

**DOI:** 10.1002/vms3.70820

**Published:** 2026-02-11

**Authors:** Tamene Bayissa, Belay Duguma, Kassahun Dessalegn

**Affiliations:** ^1^ West Wollega Agricultural and Rural Development Office Gimbi Ethiopia; ^2^ Department of Animal Sciences College of Agriculture and Veterinary Medicine Jimma University Jimma Ethiopia; ^3^ Department of Animal Science College of Veterinary Medicine and Animal Science University of Gondar Gondar Ethiopia

**Keywords:** digestible crude protein, dry matter, feed supply and demand balance, metabolizable energy

## Abstract

**Background:**

Ethiopia has the largest livestock population in Africa, making livestock crucial for food security and rural livelihoods in the country. However, the scarcity of feed, both in quantity and quality, is a major limiting factor in improving livestock productivity. The availability of major feed resources, particularly natural pastures and crop residues, is primarily influenced by agro‐ecology and season.

**Objective:**

The objective of this study was to assess feed resources and estimate the balance between feed supply and demand per year in terms of dry matter (DM), digestible crude protein (DCP) and metabolizable energy (ME) for the existing livestock in two agro‐ecological zones (AEZs) in the Haru district of Western Ethiopia.

**Methods:**

Data were collected from 174 smallholder livestock farmers (114 from ML and 60 from LL AEZs) using a semi‐structured questionnaire administered through face‐to‐face interviews. The feed balance was calculated as the difference between feed supply and livestock feed demand per year in terms of DM, ME and DCP. The data were analysed using the Statistical Package for Social Sciences version 20, with the cross‐tabulation used to examine the statistical variance of categorical data.

**Results:**

The findings revealed that natural pasture, crop residues, native grass hay, non‐conventional feed resources (NCFRs), indigenous fodder trees and shrubs (IFTSs), improved forages and crop aftermath were the main feed resources available in the study area. The annual feed DM, DCP and ME supply in ML and LL AEZs are 8.98 ± 0.09 tons and 9.68 ± 0.01 tons, 138.17 and 137.47 kg, and 54,860.841 and 61,160.841 MJ at the household level, respectively. Crop residues contributed the highest proportion (68.3% in ML and 69.5% in LL) of the total DM supply. The annual feed DM, DCP and ME requirement for maintenance of livestock was estimated to be 15.91 ± 0.58 and 13.82 ± 0.77 tons, 407.05 and 353.9 kg, and 75,914.45 and 66,003.1 MJ, respectively. The DM, DCP and ME supply satisfied only about 56.4% and 70.0%, 33.9% and 38.8%, and 72.3% and 92.7% of the annual maintenance requirement of livestock in ML and LL AEZs, respectively, showing a net deficit of 43.6% and 30%, 66.1% and 61.2%, and 27.7% and 7.3% in DM, DCP and ME per year in ML and LL AEZs, respectively. The feed deficit was higher in the ML compared to the LL AEZ, possibly due to the higher livestock population in the ML.

**Conclusion:**

The study showed that scarcity of feed supply in terms of DM, DCP and ME is a major constraint limiting livestock productivity in the study area. The feed supply showed a deficit (negative balance) of 43.6% and 30% DM, 66.1% and 61.2% DCP and 27.7% and 7.3% ME in ML and LL AEZs, respectively. The findings showed that livestock are being underfed in both AEZs of the study area. To address this, natural pasture improvement, grazing management, forage production, efficient utilization of the local conventional and NCFRs, feed conservation, urea treatment of crop residues, implementing alternative feeding practices, reducing livestock numbers and strategic supplementation are recommended to bridge the gap between feed supply and demand for sustainable livestock production in the study area.

## Graphical abstract

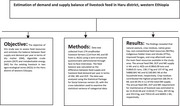

## 1 Introduction

Over many centuries, livestock has been central to the economic and social livelihoods of communities in developing countries (Hatab et al. 2019). In Ethiopia, livestock production is an important component of agriculture, contributing around 17%–25.3% to the national GDP, 39%–45% to agricultural GDP and over 50% to household income (Shapiro et al. 2017). Additionally, livestock serve as a source of draught power, food, organic manure, transportation, savings, investment, employment opportunities, insurance against emergencies, security against crop failure, for ritual purposes and other social‐cultural functions (CSA 2017). In Ethiopia, traditional farming practices in the mixed crop‐livestock farming system rely on draught oxen for land cultivation and threshing (Gebregziabher et al. 2006).

Ethiopia has the largest livestock population in Africa, with 65.3 million cattle, 39.9 million sheep, 50.5 million goats, 2.1 million horses, 8.9 million donkeys, 7.7 million camels and 59.5 million poultry (CSA 2020). Despite Ethiopia holding huge livestock population, the sector's productivity and output remain low due to various challenges. These challenges include feed scarcity in terms of both quantity and quality, a high prevalence of diseases and parasites, low genetic potential of indigenous livestock, market issues and poor access to extension, veterinary, credit, artificial insemination, marketing and infrastructure services (Duguma 2022; Duguma and Janssens 2021).

In Ethiopia, the main feed resources include natural pastures, crop residues, hay, agro‐industrial by‐products, improved forage and other sources like animal by‐products, vegetable and fruit wastes, of which the first two contribute the largest feed types (CSA 2020). However, most of these feed resources are low in quality and quantity, adversely affecting livestock productivity (Shapiro et al. 2017).

For optimal and sustainable livestock productivity, the feed resource should align with the animal population in a given area (Kechero and Geert 2014). Feed shortage, both in quality and quantity, is the most important factor limiting livestock productivity, thus hindering the economic contribution of the livestock sub‐sector in Ethiopia (Duguma and Janssens 2021; Belay et al. 2012). Discrepancies between the availability of feed resources in terms of dry matter (DM), metabolizable energy and digestible crude protein and the requirements of livestock (i.e., feed balance) indicate feed deficiencies of 9% DM, 45% ME and 42% DCP for maintenance requirements in Ethiopia (FAO 2018). In the mixed farming systems of the Ethiopian central highlands, Mekete et al. (2018) reported a 51% DM, 19% ME and 38% DCP deficiencies. In Ethiopia's central rift valley, the annual feed supply only meets 64% DM, 81% ME and 66% DCP maintenance needs of the animals per farm (Varvikko et al. 1993). Similarly, previous studies (Kechero and Geert 2014; Tadesse and Solomon 2014; Bedasa 2012; Funte et al. 2010) reported negative livestock feed balance in different regions of Ethiopia. The negative feed balance impairs livestock performance and compromises their potential role in driving economic development.

Van Soest and Robertson (1985) emphasized the importance of determining digestible protein (DCP) and metabolizable (ME), as they are the first two limiting factors for livestock productivity. To develop and implement sustainable feed improvement plans, having information on feed inventory and feed balance would be beneficial for the implementation of specific and strategic interventions (FAO 2018). Most studies have focused on evaluating feed resource availability without measuring the quantity of DM, ME and DCP obtained from each feed resource type in various parts of Ethiopia (Ayele et al. 2021).

In the Haru district, livestock production plays a significant role in the livelihoods, food security and poverty alleviation of smallholder mixed crop‐livestock farmers by providing draught power, food income, manure and savings. However, feed scarcity remains a major limiting factor for improving livestock productivity in the area. Despite this, information on feed supply and demand balance in terms of DM, CP and ME is lacking. Therefore, a proper assessment of feed supply and demand is crucial for enhancing livestock productivity. It also assists policy makers, researchers and farmers in formulating effective intervention strategies to improve feed quality and quantity for sustainable livestock production. The objective of this study was to estimate the supply and demand of feed in relation to DM, CP and ME in the midland and lowland agroecological zones of the Haru district, in Western Ethiopia.

## 2 Materials and Methods

### 2.1 Description of Study Area

The study was conducted in Haru District, West Wollega Zone of Oromia Regional State, Western Ethiopia, located 464 km west of Addis Ababa, the capital of Ethiopia. The dominant farming system in the district is crop‐livestock production. A detailed description of the study area can be found in a companion study that was previously published (Tamene et al. 2022).

### 2.2 Survey Design and Sampling

A cross‐sectional design was used in this study, implementing a multi‐stage purposive and random sampling procedure was implemented in four stages. In the first stage, the district was selected using purposive sampling based on livestock potential and AEZ variations: LL AEZ (< 1500 m above sea level) and ML AEZ (> 1500 m above sea level (MoA 2000). In the second stage, 10 *kebeles* (six from ML and four from LL AEZs) were purposively selected based on their livestock potential. *Kebele* is the lowest administrative unit in Ethiopia, next to the district. In the third stage, four *kebeles* from the ML (Genet Abo, Sadale, Yukira and Keki Adare) and two *kebeles* (Kombolcha Yonge, Alebenta) from the LL AEZ were randomly chosen for the survey. A list of 1572 households (1033 in ML and 539 in LL AEZs) keeping livestock in the six *kebeles* was provided by the local agriculture/livestock development agents’ offices. Finally, a total of 174 households (114 from ML and 60 from LL AEZs) were randomly selected from the list using the probability proportionate to sample size based on the proportion of the sampling frame in each AEZ (Cochran 1977).

(1) $$n_{0}\;\;=\frac{z^{2}\times \;\;p\;\times q}{e^{2}}=\frac{1.96^{2}\times \;\;0.15\times 0.85}{0.05^{2}}\;=195$$

(2) $$n_{1}=\frac{n_{0}}{1+\left(\frac{n_{0-1}}{N}\right)}\;=\frac{195}{1+\left(\frac{\left(195-1\right)}{1572}\right)}=174$$

Where; *n*_0_ = desired sample size according to Cochran (1977) when population greater than 10,000, *n*_1_ = finite population correction factors (Cochran 1977) when population is less than 10,000; *Z* = standard normal deviation (1.96 for 95% confidence level); *p* = 0.15 (population variability i.e., 15%); *q* = 0.85 (1‐*p*); *N* = total number of population; *e* = degree of accuracy desired (0.05); Then, 174 smallholder farmers (households [HH]) were randomly selected for interviews in the study. The number of respondents per single *kebele* was determined by the formula *W* = (A/B) × *n*_0_, where; *W* = sample size of respondents per single *kebele*; *A* = total number of households living in a single selected *kebele*; *B* = total sum of households living in all selected sample *kebeles*; *n*_0_ = sample size.

### 2.3 Data Collection

Both qualitative and quantitative data were collected through face‐to‐face interviews using a semi‐structured questionnaire with a combination of dichotomous, multiple‐choice and open‐ended questions, as well as secondary data. Additionally, focus group discussions (FGDs) were conducted with 12 members in each agroecology, along with key informant interviews and field observations. The focus group included farmers, *kebele* leaders, women, elders and local livestock extension workers. The FGDs utilized a checklist to identify major feed resources, feeding practices and major constraints faced by farmers in livestock production. The questionnaire was pretested with 14 households that were not part of the main survey, and necessary modifications were made based on the feedback from the pretesting sessions. The interviews were conducted in the local language, *Afaan Oromo*, by six survey enumerators under the close supervision of the researcher. The respondents were household heads. The questionnaire was designed to gather information on socioeconomic characteristics of respondents, livestock holding and species composition, major feed resources, land holding, types of crops produced, area cultivated for each crop type, crop yield, estimation of annual DM supply from major feed resources, Prior to the survey, all households were visited by the first author and the local livestock development/extension agents (DAs) to gather brief information about the study area, and to obtain oral informed consent from farmers regarding their willingness to participate in the study (Table 1).

**TABLE 1:** Household and sample size by agro‐ecological zone selected for interview.

Agro‐ecological zones and PAs	Total number of HHs	Proportion	Sample size
Total HHs	1572	1.00	174
Mid AEZ (*N*)	1033	0.66	114
Genet Abo PA	325	0.21	36
Sadale PA	222	0.14	25
Yukira PA	235	0.15	26
Keki Adare PA	251	0.16	28
Low AEZ (*N*)	539	0.34	60
Kombolcha Yonge	249	0.16	28
Alebenta PA	290	0.18	32

### 2.4 Estimation of Annual Dry Matter Feed Supply

The quantity of feed DM available per year from major feed resources, including crop residues, crop aftermath, improved forage and grazing lands (communal grazing land, private grazing land, fallow land and forest) was estimated using established conversion factors (multipliers) developed by FAO (1987) as presented in Table 2. The DM (tons) obtained from crop residues and grazing lands was derived from the average grain yield per area of croplands cultivated and grazing land areas from the household survey. The amount of crop residue DM collected per year by an individual farmer was quantified based on the area of land cultivated and the amount of grain produced during the cropping season. It is assumed that about 90% of the crop residue is used as feed and 10% for other purposes and wastage (Tolera and Said 1994).

**TABLE 2:** Conversion factor for estimation of DM of various feed resources in the study area.

Crop types	Conversion factor	DM yield (t/ha/year)
Barley straw	1.5	
Wheat straw	1.5	
Finger millet straw	1.5	
Maize stover	2	
Sorghum stover	2.5	
Rice straw	1.4	
Oat straw	1.5	
Teff straw	1.5	
Faba bean straw	1.2	
Chickpea straw	1.2	
Field pea straw	1.2	
**Grazing lands**		
Communal grazing land	2	
Private grazing pasture	3	
Fallow land	1.9	
Aftermath grazing	0.5	
Forest	0.7	
Bush, wood and shrub land	1.2	

*Source*: FAO (1987).

### 2.5 Estimation of Annual ME and DCP Supply

The DCP and ME requirement for maintenance of 1 TLU (weighing 250 kg) was calculated as 160 g/kg DM (FAO 1986) and 29.84 MJ (King 1983).

### 2.6 Estimation of Annual Dry Matter Requirement (Demand)

Estimates for feed requirements were based on livestock numbers. Data on the livestock numbers of the sampled households were obtained from questionnaire interviews and converted into TLU using conversion factors for cattle (0.7), sheep (0.1), goats (0.1), mules (0.7), horses (0.8) and donkeys (0.5) (Varvikko et al. 1993). The DM requirements of the livestock population were calculated based on the daily DM requirements for maintenance of 1 TLU (250 kg) consuming 2.5% of its body weight, which is 6.25 kg DM/day or 2.28 tons/year (Kearl 1982).

### 2.7 Feed Balance

Livestock feed balance at the individual household level over the entire production year was determined as the difference between the annual feed DM, ME and DCP supply estimated from major feed resources and the annual feed DM, ME and DCP demands for the average livestock holding of farmers.

### 2.8 Statistical Analysis

Data generated from this study were analysed using descriptive statistics such as frequencies, percentages, means and standard errors using the Statistical Package for Social Sciences (SPSS) software for Windows, Version 20.0 (SPSS (Statistical Procedure for Social Science) 2012, USA). Pairwise comparisons were conducted using Student *t*‐test to compare family size, farm size and livestock herd size across the two agroecological zones. Significance level was declared when *p* < 0.05.

## 3 Results and Discussion

### 3.1 Socioeconomic Characteristics of Respondents

Table 3 shows the socioeconomic characteristics of respondents in the study area. Overall, the majority (89.1%) of respondents were male, 37.4% were illiterate, and the most common age range was between 51 and 60 years (52.9%). The average family size and land sizes per household in ML and LL were 5.07 ± 0.16 persons and 3.04 ± 0.11 ha and 6.00 ± 0.19 persons and 3.16 ± 0.20 ha, respectively. The results revealed that youth involvement in livestock production was low as most of the respondents were above 50 years of age. This could be attributed to limited access of youth to resources such as land, finance and animals, leading them to frequently migrate to towns and cities for employment. Overall, about 24.71% of the surveyed farmers had an education level above primary school. Approximately 3.5% of respondents in ML had a diploma‐level education compared to none in the LL. It has been reported that well‐educated farmers have a better opportunity to acquire and process information on new technologies and improve the level of adoption of new technology than less educated ones (Kabirizi et al., 2015), and the ability of households to utilise market information and available market opportunities (Lubungu et al. 2012). The average family size reported in the present study (5.39 ± 0.13) is lower than the average of 5.86 reported by Tahir et al. (2018).

**TABLE 3:** Household socioeconomic characteristics.

Variable	ML (*N*)	ML (%)	LL (*N*)	LL (%)	Overall (*N*)	Overall (%)	*p* value
Sample size (*n*)	114		60		174		
Male	98	86	57	95	155	89.1	
Female	16	14	3	5	19	10.9	
Illiterate	41	36	24	40	65	37.4	
Reading and writing	42	36.8	21	40	63	36.2	
Primary	11	9.6	4	6.7	15	8.6	
Junior secondary	4	3.5	2	3.3	6	3.5	
High school	8	7.0	2	3.3	10	5.7	
Preparatory	2	1.8	5	8.3	7	4.0	
Certificate	2	1.8	2	8.3	4	2.3	
Diploma	4	3.5	0	0	4	2.3	
Age 30–40	25	14.4	6	3.4	31	17.8	
Age 41–50	30	17.2	9	5.2	39	22.4	
Age 51–60	51	29.3	41	23.6	92	52.9	
Age ≥ 61	8	4.6	4	2.3	12	6.9	
Family size (mean ± SE)	114	5.07 ± 0.16	60	6.00 ± 0.19	174	5.39 ± 0.13	0.00
Land holding (ha) (mean ± SE)	114	3.04 ± 0.11	60	3.16 ± 0.20	174	3.04 ± 0.11	0.00

### 3.2 Livestock Herd Size and Species Composition

Livestock herd size in tropical livestock unit (TLU) and structure per household in the study area are presented in Table 4. Before assessing feed supply and demand, it is important to quantify the livestock population kept in the study area. All the surveyed farmers kept local livestock species. The overall mean livestock herd/flock size per household obtained in this study differed significantly between the two AEZs (*p* < 0.05). The average livestock holding was 6.9 ± 0.26 and 6.1 ± 0.34 TLU per household in the ML and LL AEZs, respectively, with an overall average of 6.7 ± 0.21 TLU.

**TABLE 4:** Mean ± SE of livestock herd size and structure (TLU) per household in the ML and LL AEZs of Haru district.

Livestock species	Mean herd size and structure			
	ML (*n* = 114)	LL (*n* = 60)	Overall (*n* = 174)	*p* value
Cattle	4.83 ± 0.23^a^	3.89 ± 0.28^b^	4.5 ± 0.18	0.013
Goats	0.21 ± 0.03^b^	0.29 ± 0.04^a^	0.24 ± 0.03	0.045
Sheep	0.42 ± 0.03^a^	0.32 ± 0.04^a^	0.39 ± 0.02	0.983
Donkeys	0.44 ± 0.04^a^	0.48 ± 0.06^a^	0.45 ± 0.04	0.058
Mules	0.2 ± 0.03^a^	0.05 ± 0.03^a^	0.15 ± 0.02	0.305
Overall	6.97 ± 0.26^a^	6.06 ± 0.34^b^	6.66 ± 0.21	0.001

*Note*: Means with different superscripts within the rows between AEZ are significantly different at *p* < 0.05.

Abbreviation: SE, standard error of means.

In both AEZs, cattle were the dominant livestock species kept by the interviewed farmers. This could be due to the multiple roles of cattle as a source of milk, meat, draught power, income and manure. According to the surveyed farmers, the food security and livelihood of their household depend on owning at least a pair of oxen for draught power. The cattle herd size per household was 4.83 ± 0.23 and 3.89 ± 0.28 for ML and LL, respectively, with an overall average of 4.5 ± 0.18 TLU. Cattle herd size showed a significant difference between the AEZs (*p* < 0.01), with a larger herd size in ML AEZ than in LL AEZ. This might be attributed to the high prevalence of trypanosomiasis and inadequate access to veterinary services in the LL AEZ. In addition, anthrax, pasteurellosis, internal and external parasites and foot‐and‐mouth disease were reported to be common animal health problems in the LL AEZ. The average cattle herd size obtained in the present study is lower than the findings of Belay and Negesse (2019), who reported mean values of 8.19 ± 0.95 and 4.57 ± 0.95 for ML and LL, respectively.

The mean sheep flock size per household was 0.42 ± 0.03 and 0.32 ± 0.04 for ML and LL, respectively, with no significant difference across AEZs (*p* > 0.05). The relatively higher number of sheep in HL might be due to their better adaptation to cool climatic conditions. The average number of sheep observed in this study is lower than the results of Belay and Negesse (2019), who reported an average sheep herd size of 0.50 ± 0.30 and 0.45 ± 0.21 in ML and LL, respectively.

The average flock size of goats per household was 0.21 ± 0.031 and 0.29 ± 0.04 for ML and LL, and varied significantly across AEZs (*p* < 0.05). Farmers in the LL AEZ had more goats than in the ML AEZ. This may be due to the better adaptability of goats to hot climatic conditions and the abundance of browsing vegetation in the LL compared to the ML AEZ. The results of this study are in agreement with the findings of Belay and Negesse (2019), who reported higher numbers of goats (1.34 ± 0.23) in LL than in ML (1.00 ± 0.57) in the Burie Zuria district in Northwestern Ethiopia. The variation observed in livestock herd size between the present findings and literature may be due to differences in agroecology, grazing land availability and the animal production purpose of farmers.

There was no difference in the number of donkeys and mules between the AEZs (*P *> 0.05). Additionally, the results showed that the number of female breeding cattle, goats and sheep was higher than that of males. This could be attributed to the practice of keeping females for breeding in order to ensure the sustainability of production by providing future replacement animals. Respondents indicated that breeding females are only sold when they are old and no longer able to reproduce. The high number of oxen compared to cows can be explained by their importance as a source of draught power. Respondents indicated that oxen are only sold when they are too old and no longer useful for draught power purposes.

### 3.3 Estimated Annual Feed DM Supply From Major Feed Resources

Table 5 shows the estimated annual supply of feed DM supply obtained from major feed resources for the maintenance requirements of livestock in the study area. The total quantity of DM (tons/year) was estimated from various feed sources available in the study area, including natural pastures, crop residues, crop aftermath, fallow land, improved forages and forest land grazing.

**TABLE 5:** Estimated DM supply (tons/ household/year) from major feed types in the ML and LL AEZs in Haru district.

Feed resources	ML (*N* = 114)	% Share	LL (*N* = 60)	% Share	Overall (*N* = 174)	% Share	*p* value
Crop residues	6.13 ± 0.25^a^	68.3	6.73 ± 0.37	69.5	6.43 ± 0.21	68.9	0.170
Crop aftermath	1.41 ± 0.21^a^	15.7	1.13 ± 0.18^b^	11.7	1.31 ± 0.15	13.7	0.000
Private pasture	0.57 ± 0.04^b^	6.3	0.77 ± 0.06^a^	7.9	0.64 ± 0.04	7.1	0.011
Communal pasture	0.24 ± 0.03^b^	2.7	0.59 ± 0.05^a^	6.1	0.36 ± 0.03	4.4	0.000
Fallow land	0.32 ± 0.04	3.6	0.21 ± 0.06	2.2	0.28 ± 0.03	2.9	0.125
Improved forage	0.16 ± 0.02	1.8	0.15 ± 0.02	1.5	0.15 ± 0.02	1.7	0.720
Forest land	0.15 ± 0.02	1.7	0.10 ± 0.03	1.0	0.13 ± 0.02	1.3	0.125
Total supply	8.98 ± 0.09	100	9.68 ± 0.01	100	9.33 ± 0.07	100	

*Note*: Means with different superscripts in the rows between AEZ are significantly different at *p* < 0.05.

Abbreviations: DM, dry matter; SE, standard error of means.

The total DM supply from all sources was estimated to be 8.98 ± 0.09 tons per year in ML and 9.68 ± 0.01 tons/year in the LL AEZs, with an overall average of 9.3 ± 0.07 tons per year. Crop residues contributed the highest total feed DM supply of 68.3% and 69.5%, followed by aftermath at 11.7% and 14%, private pasture at 6.3% and 7.9%, communal pasture at 2.7% and 6.1%, fallow at 3.6% and 2.2%, improved forage at 1.8% and 1.5%, and forest land at 1.7% and 1% in ML and LL AEZs, respectively.

In the ML, the DM obtained from crop residue, stubble grazing, private grazing land, fallow land, communal grazing land, improved forages and forest land grazing were 6.13 ± 0.25, 1.41 ± 0.21, 0.57 ± 0.04, 0.32 ± 0.04, 0.24 ± 0.03, 0.16 ± 0.02 and 0.15 ± 0.02 tons/year, respectively. Crop residues contributed the highest (68.92%) to the total annual feed DM supply, followed by crop stubble, while the least DM was obtained from improved forages. The high DM yield from crop residues could be attributed to the expansion of cropland at the expense of grazing lands.

In the LL AEZ, the DM obtained from crop residue, stubble grazing, private grazing land, communal grazing land, fallow land, improved forages and forest grazing was estimated to be about 6.73 ± 0.37, 1.13 ± 0.18, 0.77 ± 0.06, 0.59 ± 0.05, 0.21 ± 0.06, 0.15 ± 0.02 and 0.10 ± 0.03 tons/year, respectively. The findings of the present study did not agree with the results of Ayele et al. (2021), who reported that natural pasture followed by crop residues in the ML and crop residues followed by natural pasture in the LL contributed the highest DM to the total feed supply.

The estimated annual DM from private grazing, communal grazing and stubble grazing showed a significant difference (*p* < 0.05) across the two AEZs. This difference could be attributed to the availability of more communal grazing land and lower human population density in the LL AEZ compared to the ML AEZ, as well as higher crop production in ML than in LL. Forest land grazing contributed the least to the annual DM supply compared to the other feed resources in the surveyed area. During FGDs participants mentioned that they allocated more land to cereal and coffee production than to grazing land in order to meet household food security needs and generate cash income.

### 3.4 Feed Balance (Supply and Requirement)

Table 6 presents the balance between feed availability/supply and demand/requirements in terms of DM, ME and DCP across the two AEZs in the study area. The results revealed that the available feed balance was negative, indicating a critical feed shortage to satisfy the DM, ME and DCP requirements for the prevailing livestock during the study year.

**TABLE 6:** Average estimated annual supply and demand balance of DM, ME and DCP for maintenance requirements of ruminants by AEZ in Haru district.

Feed nutrients	Annual supply	Annual requirements	Feed deficit	% Satisfied
**ML AEZ (*N* = 114)**				
DM (mt/hh/year)	8.98 ± 0.09	15.91 ± 0.58	−6.93 ± 0.71	56.4
DCP (kg/hh/year)	138.17	407.05	−268.89	33.9
ME (MJ/hh/year)	54,860.841	75,214.45	−21,053.61	72.3
**LL AEZ (*N* = 60)**				
DM (mt/hh)	9.68 ± 0.01	13.82 ± 0.77	−4.14	70.0
DCP (kg/hh/year)	137.47	353.9	−216.43	38.8
ME (MJ/kg/hh/year)	61,160.841	66,003.1	−4842.3	92.7
**Overall**				
DM (mt/hh/year)	9.33 ± 0.07	15.19 ± 0.47	−5.86	63.2
DCP (kg/hh/year)	137.82	388.94	−251.12	36.3
ME (MJ/hh/year)	57,159.1	72,538.1	−15,379.0	82.5

Abbreviations: DCP, digestible crude protein; DM, dry matter; ME, metabolizable energy; TLU, Tropical livestock unit.

The estimated DM, DCP and ME per year from major feed resources in the ML AEZ were approximately 8.98 ± 0.09 tons, 138.17 kg and 54,860.8 MJ, respectively. The requirements, however, were 15.91 ± 0.58 tons, 407.05 kg and 75,914.45 MJ, respectively, resulting in a deficit of about −6.93 ± 0.71 tons of DM, −268.89 kg of DCP and −21,053.61 ME, respectively. Therefore, in the ML AEZ, the available feed resources only satisfied about 56.4%, 33.9% and 72.3% of the annual DM, DCP and ME maintenance requirements of the livestock, respectively. This indicates the need for supplementation of animals with both energy and protein sources of feed. The negative feed balance observed in the current study is consistent with the findings of Ayele et al. (2021), who reported that the DM, DCP and ME supply satisfied only about 77.56% and 56.73%; 29.10% and 23.10%; and 102.34% and 74.86% of the livestock demand per year in ML and LL AEZs in Lalo Kile district. However, the variation in percent of supply might be explained by differences in feed resources availability, feed quality and livestock population.

In the LL AEZ, the estimated annual DM, DCP and ME from various feed resources were approximately 9.68 ± 0.01 tons, 137.47 kg and 61,160.841 ME, respectively. The requirements are about 13.82 ± 0.77 tons, 353.9 kg and 66,003.1 MJ, respectively, resulting in a negative balance of approximately −4.14 tons, −216.43 kg and −4842.3 ME, respectively. Therefore, the available feed resources only satisfied around 70.0%, 38.8% and 92.7% of the annual DM, DCP and ME maintenance requirements, respectively. In the mixed farming systems of the Ethiopian central highlands, Mekete et al. (2018) reported a 51% DM, 19% ME, and 38% DCP deficiencies. In Ethiopia's central rift valley, the annual feed supply only meets 64% DM, 81% ME, and 66% DCP maintenance needs of the animals per farm (Varvikko et al. 1993). The differences observed between the findings of the current study and previous works regarding feed balance could be attributed to variation in feed resources availability and the number of livestock considered.

Overall, the DM, DCP and ME gap was higher in the ML compared to the LL. This difference could be attributed to the large livestock population and decreasing grazing land areas in the ML due to the expansion of crop production, as opposed to the LL AEZ. The total annual usable DM and ME supply recorded in this study was lower than the findings of Ayele et al. (2021), who reported 12.580 ± 22 and 11.170 ± 46 tons and 70371 MJ and 79254 MJ in ML and LL AEZs, respectively. Additionally, the supply of DCP (116.41 kg in ML and 120.81 kg in LL) was lower than the present findings. The negative feed balance observed in the current study is consistent with previous findings under smallholders' mixed farming systems in other regions of Ethiopia (Kassa et al. 2003; Amsalu and Addisu 2014; Kechero and Geert 2014).

## 4 Conclusion

Based on the results of this study, natural pastures, crop residues, crop aftermath, fallow land, improved forages and forest land grazing were identified as the main feed resources in the surveyed area. The estimation of available feed resources in terms of DM, DCP and ME revealed a deficit in feed supply within the district. In ML, the estimated supply of DM was 8.98 ± 0.09 tons, DCP was 138.17 kg and ME was 54860.841 MJ, while the estimated requirements were 15.91 ± 0.58 tons, 407.05 kg and 75,914.45 MJ, respectively. This only covers approximately 56.4% of DM, 33.9% of DCP and 72.3% of MJ of the requirement. In the LL AEZ, the estimated supply of DM was 9.68 ± 0.01 tons, DCP was 137.47 kg and ME was 61,160.841 MJ, while the estimated requirements were 13.82 ± 0.77 tons, 353.9 kg and 66,003.1 MJ, respectively. This supply covers only about 70%, 38.8% and 92.7% of the total annual requirements, respectively. Overall, the scarcity of DM, DCP and ME is the major factor limiting livestock productivity in the area. To address the feed supply deficit and ensure the sustainability of smallholder livestock production in Haru district, it is recommended to focus on strengthening pasture management, forage production, feed conservation, efficient use of available conventional and unconventional feed resources, integrating forages with food crops, proper collection, storage and treatment of crop residues, adjusting livestock numbers based on available feeds, implementing alternative feeding methods and supplementing with energy and protein‐rich feed sources, especially during the dry season.

### 4.1 Limitations of the Study

The authors acknowledge limitations in the data used for this study, particularly in terms of the use of qualitative inquiry. Farmers’ perspectives were gathered through a household questionnaire, relying on farmer recall, secondary data, estimations and lacking measurement or laboratory analysis of feeds. These factors could introduce bias and may not be entirely accurate. But they are not uncommon limitations in similar survey studies. Despite these limitations, this study offers interesting viewpoints and useful information on feed supply and demand balance in the study area.

## Author Contributions

**Tamene Bayisa**: conceptualization, methodology, data collectition, investigation, formal analysis, data curation and inteprated data, and drafted the manuscript. **Belay Duguma**: concepptualization, methodology, data curation, supervision, revised and edited the manuscript. **Kassahun Dessalegne**: conceptualization, methodology, data curation, supervision.

## Funding

There is no special funding for this work. However, Jimma University College of Agriculture and Veterinary Medicine provided minimal financial support for enumerators for data collection for MSc thesis research of the first author.

## Ethics Statement

The authors affirm that the journal's ethical rules, as stated on the author guidelines page, were followed. However, the study did not use animals, but rather a questionnaire survey. As a result, data collection was carried out following a verbal agreement with the study's target farmers. The protocol was approved by the Jimma University College of Agriculture and Veterinary Medicine Ethical Approval committee in accordance with the Jimma University research guidelines and regulations. Verbal informed consent was obtained from all participants, who were thoroughly informed about the objectives of the study, procedures, content of the schedule used for data collection, and their rights, including the option to withdraw at any time. Written consent was not requested due to varying levels of literacy among participants and a distrust of the signed consent form. They were assured that their identity would be kept confidential. Informed verbal consent was also given by each respondent for publication of the results in an online‐access journal.

## Conflicts of Interest

The authors declare no conflicts of interest.

## Data Availability

The data used to support the findings of this study are included within the article.
